# Regulator of G-Protein Signaling 19 (RGS19) and Its Partner Gα-Inhibiting Activity Polypeptide 3 (GNAI3) Are Required for zVAD-Induced Autophagy and Cell Death in L929 Cells

**DOI:** 10.1371/journal.pone.0094634

**Published:** 2014-04-21

**Authors:** Ting Wu, Yuanyue Li, Deli Huang, Felicia Han, Ying-Ying Zhang, Duan-Wu Zhang, Jiahuai Han

**Affiliations:** 1 Department of Basic Medical Sciences, Cancer Research Center, Medical College, School of Life Sciences, Xiamen University, Xiamen, Fujian, China; 2 State Key Laboratory of Cellular Stress Biology, Innovation Center for Cell Biology, School of Life Sciences, Xiamen University, Xiamen, Fujian, China; Yong Loo Lin School of Medicine, National University of Singapore, Singapore

## Abstract

Autophagy has diverse biological functions and is involved in many biological processes. The L929 cell death induced by the pan-caspase inhibitor benzyloxycarbonyl-Val-Ala-Asp-(OMe)-fluoromethyl ketone (zVAD) was shown to be an autophagy-mediated death for which RIP1 and RIP3 were both required. It was also reported that zVAD can induce a small amount of TNF production, which was shown to be required for zVAD-induced L929 cell death, arguing for the contribution of autophagy in the zVAD-induced L929 cell death. In an effort to study RIP3 mediated cell death, we identified regulator of G-protein signaling 19 (RGS19) as a RIP3 interacting protein. We showed that RGS19 and its partner Gα-inhibiting activity polypeptide 3 (GNAI3) are involved in zVAD-, but not TNF-, induced cell death. The role of RGS19 and GNAI3 in zVAD-induced cell death is that they are involved in zVAD-induced autophagy. By the use of small hairpin RNAs and chemical inhibitors, we further demonstrated that zVAD-induced autophagy requires not only RIP1, RIP3, PI3KC3 and Beclin-1, but also RGS19 and GNAI3, and this autophagy is required for zVAD-induced TNF production. Collectively, our data suggest that zVAD-induced L929 cell death is a synergistic result of autophagy, caspase inhibition and autocrine effect of TNF.

## Introduction

Programmed cell death plays an essential role in developmental and pathophysiological processes. The dysregulation of cell death contributes to disorders, including autoimmune diseases, neurodegenerative diseases, ischemia-reperfusion damage and cancer. The processes of the most commonly-observed types of programmed cell death include apoptosis, necroptosis and autophagy-mediated cell death. Since different processes can be dominant in either different cell lines or in the same cell lines under different simulations, there is an obvious necessity to clarify the yet largely unknown relationship among those types of cell death.

Tumor necrosis factor-α (TNF) is a pleiotropic cytokine which induces either apoptosis or necroptosis depending on cell types as well as conditions of stimulation [1], [2], [3]. The level of RIP3 expression appears to be a crucial determinant for the choice of apoptosis or necroptosis [4], [5], [6]. Without RIP3 expression, cells undergo apoptosis when stimulated with TNF, whereas high level of RIP3 expression can convert TNF-induced apoptosis to necrotic cell death. Current model of TNF-induced apoptosis and necroptosis is that: TNF and TNF receptor 1 (TNFR1) engagement leads to formation of complex I by recruiting several effectors/adaptors including RIP1. Complex I triggers NF-κB and mitogen activated protein (MAP) kinase activation. Under conditions such as deubiqutination of RIP1, complex II containing FADD, Caspase-8 and RIP1 forms and initiates apoptosis. When RIP3 is present, incorporation of RIP3 into complex II results in the formation of necrosome (also called complex IIb) and the cell dies via necroptosis [7]. Caspase-8 can cleave RIP1, RIP3 and other pro-necrosis proteins and thus has an inhibitory effect on necroptosis. Pan caspase inhibitor benzyloxycarbonyl-Val-Ala-Asp (OMe)-fluoromethylketone (zVAD) is not only widely used to block apoptosis but also commonly applied to enhance necroptosis due to its inhibitory effect on caspase-8. However, zVAD exerts its function on cell death not simply via caspase inhibition. zVAD by itself can induce cell death in certain cell lines such as L929 [8]. It was originally reported that zVAD-induced cell death is autophagy-mediated because inhibition of autophagy blocks zVAD-induced cell death [8], [9]. Another study showed that c-Src-dependent activation of JNK and ERK is involved in zVAD-induced cell death [10]. However, conflicting data on whether inhibition of autophagy can block zVAD-induced L929 cell death was also reported [11], [12]. There is evidence that zVAD-induced cell death requires autocrine of zVAD-induced secretion of TNF. PKC-MAPKs-AP-1 pathway was shown to play a role in zVAD-induced TNF production [13].

Guanine nucleotide-binding proteins (G-proteins) are a family of signal mediators that are essential for a variety of cellular functions [14], [15], [16]. Their activities are regulated by factors that control their ability to bind to and hydrolyze GTP to GDP. Heterotrimeric G protein complexes are made up of α, β and γ subunits. There are many classes of G_α_ subunits including Gα_s_ (G stimulatory) and Gα_i_ (G inhibitory). Different classes of Gα behave differently in the recognition of the effectors, but they share a similar mechanism of activation. The Gα_i_ family consists of three closely related members, Gα_i1–3_ (also named GNAI1-3) that is characterized by their sensitivity to pertussis toxin (PTx). The GNAI1-3 isoforms share 85–95% of amino acid sequence identity and overlapping expression patterns. Previous studies suggested the roles of these GNAI isoforms in distinct cellular responses. GNAI3 is required for autophagy at the sequestration step in human colon cancer cell line HT-29 [17], [18] but GNAI3 deficiency in mouse was also found to enhance the autophagic proteolysis induced by liver perfusion [19].

Regulators of G-protein signaling (RGS) are GTPase-accelerating proteins that promote GTP hydrolysis by α subunit of heterotrimeric G proteins. All RGS proteins contain an RGS-box (or RGS domain) that is required for their activities. Some RGS proteins contain additional domains that may confer the interaction of a given RGS to other proteins or additional functionality. RGS19 (also known as Gα-interacting protein, GAIP) is known to interact with GNAI3 [20] and was shown to promote the macroautophagic pathway [18].

RIP3 is required in both TNF-induced necroptosis and zVAD-induced autophagic cell death [3]. There should be specific proteins for the regulation of RIP3-mediated necroptotic and autophagy-mediated cell death. RGS19 is one of the proteins in the immunoprecipitates of RIP3 identified by our mass spectra analysis. We show here in this report that RGS19 and GNAI3 are involved in zVAD-, but not TNF-induced L929 cell death. RGS19 and GNAI3 are both required for zVAD-induced autophagy. zVAD-induced autophagy and TNF secretion appear to work together in triggering autophagy-mediated death of L929 cells.

## Materials and Methods

### Reagents

Murine TNF-α was bought from eBioscience (San Diego, CA, USA). zVAD was from R&D Systems (Minneapolis, MN, USA). Necrostatin-1(Nec-1) and propidium iodide (PI) were purchased from Sigma (St. Louis, MO, USA). The rabbit polyclonal antibodies against RIP3 were generated as described in our previous paper [6]. Mouse anti-RIP1 antibodies were from BD Pharmingen (San Diego, CA, USA). Mouse anti-β-actin antibodies were purchased from Santa Cruz Biotechnology (Santa Cruz, CA, USA). Rabbit anti-p-ERK, rabbit anti-ERK, rabbit anti-p38, rabbit anti-p-p38, rabbit anti-p-JNK and rabbit anti-PI3KC3 (D9A5) antibodies were obtained from Cell Signaling (Danvers, MA, USA). Rabbit anti-JNK antibody was from Proteintech (Wuhan, China). Mouse anti-Flag antibodies (M2) were obtained from Sigma (Saint Louis, MO). Chemical inhibitors are: ERK inhibitor PD98059 (EMD Millipore), JNK inhibitor SP600125 (EMD Millipore), p38 inhibitor SB203580 (Sigma), and PI3K inhibitor LY294002 (EMD Millipore).

### Cells and Cell Culture

Mouse fibrosarcoma L929 cells were bought from American Type Culture Collection (ATCC). zVAD insensitive L929 subline is an isolated single clone from the pool of L929 cells. All cells were cultured in Dulbecco’s Modified Eagle’s Medium (Invitrogen) supplemented with 10% fetal bovine serum (Gibco), 2 mM L-glutamine, 100 units/ml penicillin and 100 µg/ml streptomycin, at 37°C in the humidified incubator containing 5% CO_2_.

### Cell Viability Studies

The viability of cells was determined by flow cytometry with two parameters: plasma membrane integrity and cell size. The plasma membrane integrity was judged by the ability of cells to exclude PI and the cell size was evaluated by forward-angle light scattering. Briefly, after treatment, cells were trypsinized, collected by centrifugation at 1,000×g, washed once with PBS, and resuspended in PBS containing 5 µg/ml PI. The level of PI incorporation was quantified on a flow cytometer (EPICS XL; Beckman Coulter, Fullerton, CA, USA). Viable cells are PI-negative with a normal size.

### shRNA Constructs

For shRNA construct, shRNA oligo was cloned into a lenti-based shRNA vector pLV-RNAi (from Biosettia, San Diego, CA, USA) according to the manufacturer’s instructions. The sequences of shRNA oligos for mouse RGS19, GNAI3, TNFR1 and TNF were as follows: RGS19-1 (5′-GCCTCTGTTTATCCCACAA-3′), RGS19-2 (5′-GCCCTAAGGAAGTACAGAG-3′), RGS19-3 (5′-GGCTTGTGAGGAGTTGAAA-3′), GNAI3-1 (5′-GGAAATGAACCGAATGCAT-3′), GNAI3-2 (5′-GGGAATGTCTAATTGGCAT-3′), GNAI3-3 (5′-GCTTGGACTCTGTAATCTA-3′), TNFR1-1 (5′-GCTAGGTCTTTGCCTTCTA-3′), TNFR1-2 (5′-GAAGTCTACTCCATCATTT-3′), TNFR1-3 (5′-GCCTCGTGCTTTCCAAGAT-3′), TNF-1 (5′-GGGATGAGAAGTTCCCAAA-3′), TNF-2 (5′-GCCGATTTGCTATCTCATA-3′), TNF-3 (5′-GGTCTACTTTGGAGTCATT-3′). The sequences of shRNA oligos for mouse RIP3, RIP1, and PI3KC3 were referred to in our previous study [3]. A non-target shRNA oligonucleotide (5′-CAACAAGATGAAGAGCACCAA-3′) was used as a negative control. All constructs were verified by DNA sequencing.

### Lentiviral Production and Infection

For lentiviral production, the expression construct or shRNA plasmid plus the virus packaging plasmids pMDL g/p RRE, pRSV-REV, and pVSV-G (Invitrogen) were cotransfected into HEK 293T cells using the calcium phosphate method. Cell medium was changed 12 h after transfection, and viral supernatants were collected 36 h later. The cell-free viral supernatants were filtered using 0.45-µm syringe filters, and polybrene (Sigma) was added to a final concentration of 8 µg/ml prior to infection of target cells. The cells were then centrifuged at 2,500 rpm for 30 min and returned to the incubator immediately.

### Co-immunoprecipitation

Myc-GNAI3 with Flag-RIP3 or Flag-RGS19, Flag-RIP3 with Myc-RGS19 were respectively co-expressed in 293T cells, Flag-RIP3 or Flag-RGS19 was then immunoprecipitated with anti-Flag M2-beads, and Myc-GNAI3 or Myc-RGS19 was detected in the immunoprecipitates with anti-myc antibody by Western blotting.

### Western Blotting

Cells were washed twice with PBS and lysed in 1× SDS sample buffer (60 mM Tris-Cl, pH 6.8, 10% Glycerol, 2% SDS, 5% β-mercaptoethanol, 0.01% bromphenolblue). The samples were then sonicated on ice to fragment the genomic DNA, and incubated at 95–100°C for 10 min. After centrifugation, the samples were subjected to 10% SDS-PAGE gels. Following electrophoresis, proteins in gels were transferred to PVDF membranes and the blocked membranes were blotted with antibodies. The chemiluminescent signals were detected by using an ECL substrate kit (Pierce).

### Measurement of Autophagic Flux

Cells were cultured with chloroquine (25 µM) and treated with or without zVAD (20 µM) for 12 hours. Cells were then harvested, lysed, normalized for total protein content, and subjected to immunoblot analysis using anti-LC3 antibody. Autophagic flux was measured by comparison of the levels of LC3-II after zVAD treatment with or without chloroquine treatment.

### Real-time PCR Analysis

2 µg total RNA was used to prepare cDNA using oligo(dT)12 as a primer. The SYBR green PCR Master Mix kit (Applied Biosystems) was used for real-time PCR analysis. Each sample was run in triplicate. The relative amounts of RNA were calculated with the ΔΔCt method and normalized with an internal control, glyceraldehydes-3-phosphate dehydrogenase (GAPDH).

### ELISA Assay

Cell culture supernatants were collected and concentrated 10 folds by Amicon Ultra filter units (Millipore). TNF concentration was measured by ELISA kit (R&D) according to the instruction from the manufacturer. Briefly, 50 µL of Assay Diluent was added to each well, and then 50 µL of Standard, Control, or sample was added. The mixtures were incubated at room temperature for 2 hours. After aspiration and wash, 100 µL of Conjugate was added and was incubated at room temperature for 2 hours. Then aspiration and wash was performed. 100 µL Substrate Solution was added to each well and incubated at room temperature for 30 minutes. 100 µL of Stop Solution was added to stop the reactions and the absorbance of the samples was read at 450 nm wavelength.

### Statistical Analysis

All values in the figures are expressed as the mean ± s.d. of triplicates. The statistical significance of the differences between two groups was determined using the Student’s *t* test.

## Results

### RGS19 and its Partner GNAI3 can Interact with RIP3 and are Required for zVAD-, but not TNF-, Induced Cell Death

**Figure 1:**
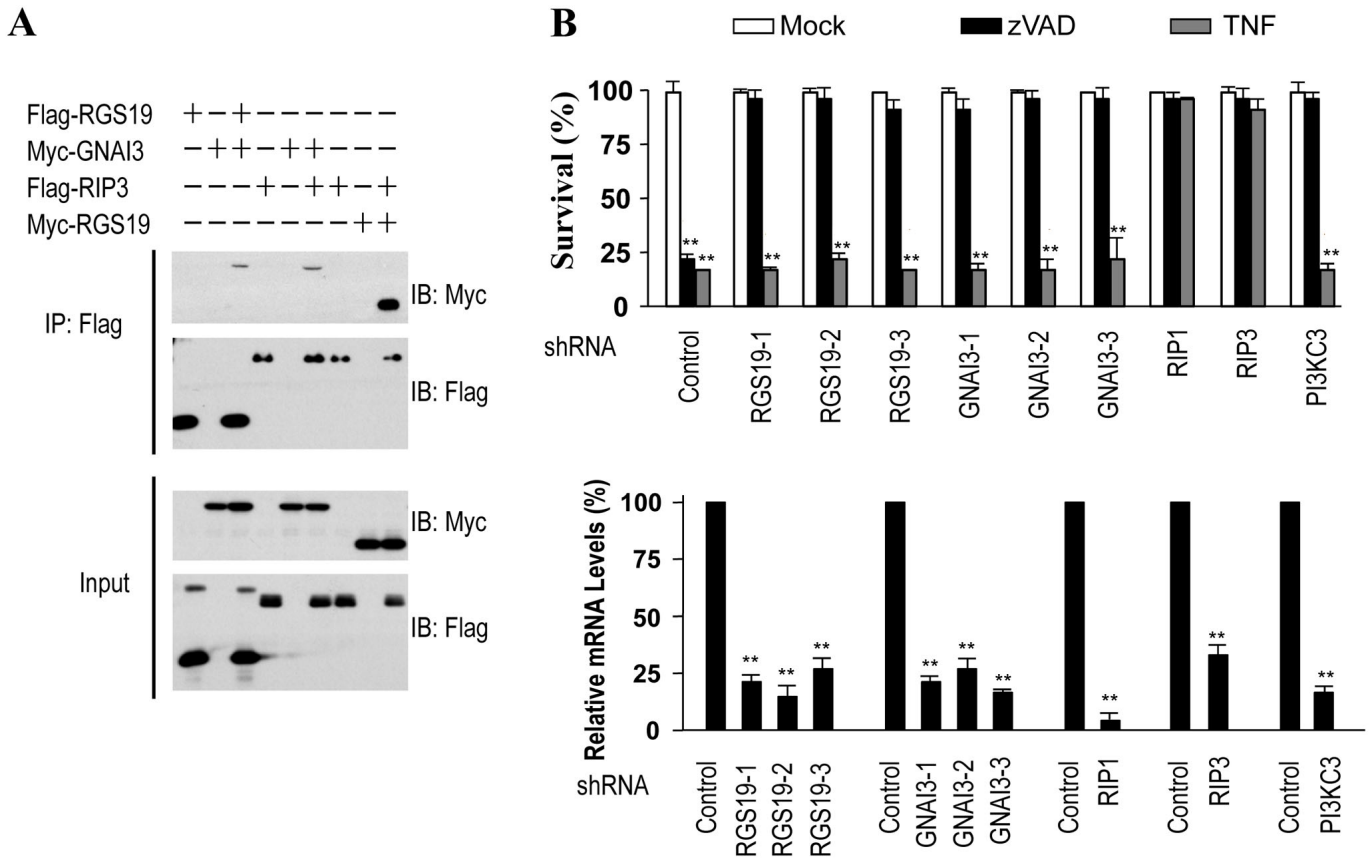
RGS19 and GNAI3 interact with RIP3 and promote zVAD- but not TNF-induced cell death. (**A**) Co-IP analysis is used to study the interactions between RGS19 and RIP3, GNAI3 and RIP3, RGS19 and GNAI3. Myc-GNAI3 with Flag-RIP3 or Flag-RGS19, Flag-RIP3 with Myc-RGS19 were respectively co-expressed in 293T cells, Flag-RIP3 or Flag-RGS19 was then immunoprecipitated with anti-Flag M2-beads, and Myc-GNAI3 or Myc-RGS19 was detected in the immunoprecipitates with anti-myc antibody by Western blotting. (**B**) Knockdown of RGS19 or GNAI3 effectively blocked zVAD-induced cell death, but had no effect on TNF-induced cell death. Control and RGS19- or GNAI3-knockdown L929 cells were treated for 24 h with mock, TNF (10 ng/ml) or zVAD (20 µM). RIP1-, RIP3- or PI3KC3-knockdown L929 cells were also included. Then cell viabilities were measured (upper panel). The knockdown efficiencies were determined by relative mRNA levels calculated from real-time PCR results (lower panel). **, p<0.01.

In searching for the interacting proteins of RIP3, we analyzed the immunoprecipitates of RIP3 by mass spectrometry (MS) and found RGS19. We co-expressed Flag-RIP3 and Myc-RGS19 in 293T cells to determine whether RGS19 can directly interact with RIP3. Flag-RIP3 was then immunoprecipitated with anti-Flag antibody, and Myc-RGS19 was detected in the immunoprecipitates by Western blotting (Figure 1A). Thus, RGS19 can interact with RIP3. Since RGS19 is known to interact with and negatively regulate GNAI3, α subunit of Gi3 [20], [21], we analyzed whether GNAI3 can interact with RIP3. We co-expressed Myc-GNAI3 with Flag-RIP3 or Flag-RGS19 in 293T cells and analyzed their interaction by co-immunoprecipitation. As shown in Figure 1A, RGS19 interacted with GNAI3 as expected and also interacted with RIP3.

Considering RIP3’s role in necroptosis, we examined whether RGS19 or GNAI3 plays any role in cell death. We delivered lentivirus expressing small hairpin RNAs (shRNAs) targeting RGS19 or GNAI3 into L929 cells. We were able to effectively knockdown RGS19 and GNAI3 each by three different shRNAs (Figure 1B, lower panel). We treated RGS19 and GNAI3 knockdown cells with zVAD or TNF and examined cell death. Knockdown of RGS19 or GNAI3 significantly blocked zVAD-induced cell death, but these depletions had no effect on TNF-induced cell death (Figure 1B, upper panel). Within the control groups, consistent with our published study [3], knockdown of RIP1 or RIP3 blocked both zVAD- and TNF-induced cell death; knockdown of PI3KC3, an essential kinase for autophagy, inhibited the zVAD-, but not TNF-, induced cell death.

### RGS19 and GNAI3 are Involved in zVAD- and TNF-induced Autophagy

**Figure 2:**
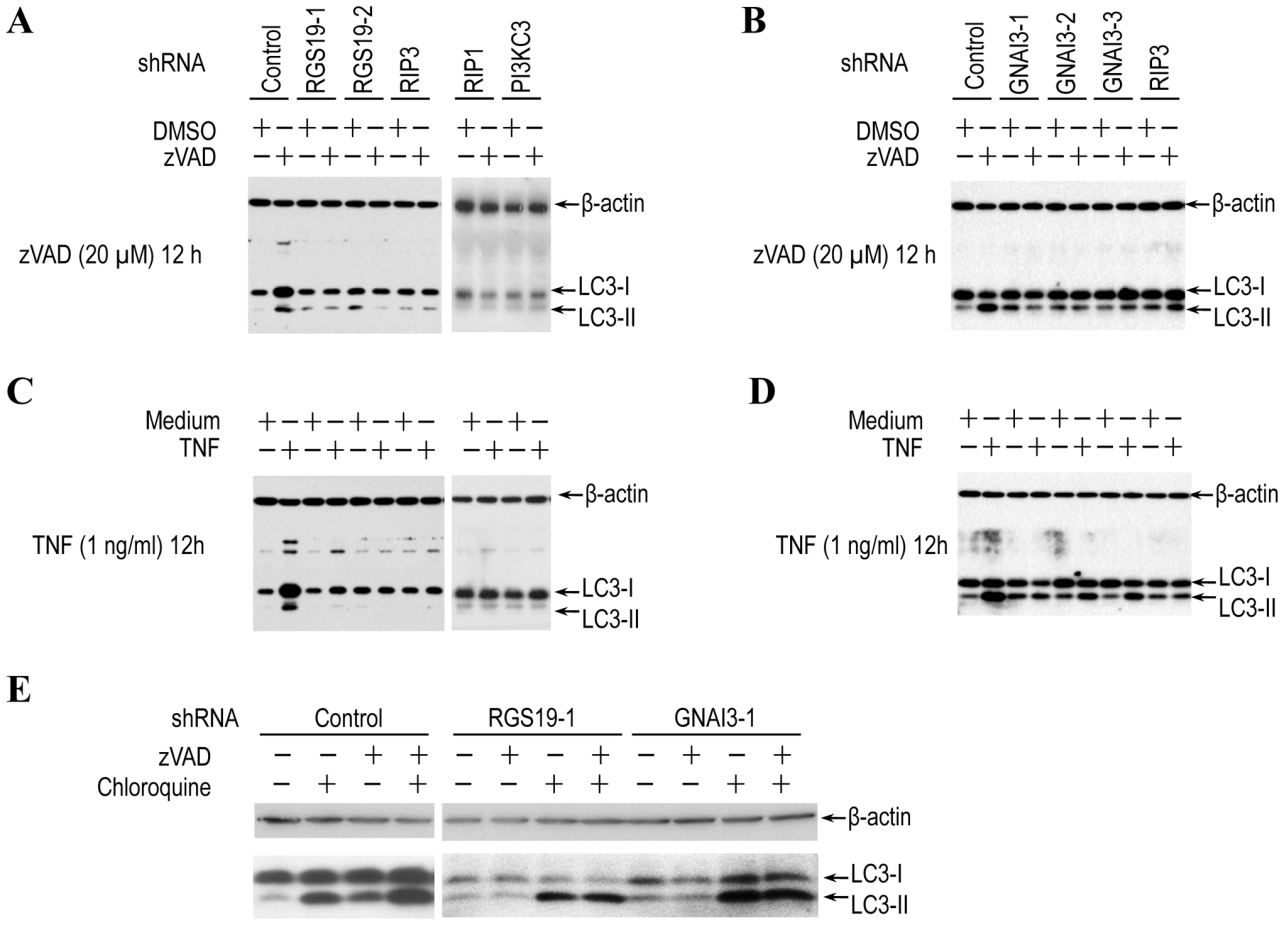
Knockdown of RGS19 or GNAI3 inhibits both zVAD- and TNF-induced autophagy. (**A–B**) Knockdown of RGS19 or GNAI3 inhibited zVAD-induced modification of LC3. Control and RGS19-knockdown (**A**) or GNAI3 knockdown (**B**) L929 cells were treated with mock or zVAD (20 µM) for 12 h. Cell lysates were subjected to western blot analysis with antibodies against LC3 and β-actin. Results from RIP1- or PI3KC3-knockdown cells were included as controls. (**C–D**) Knockdown of RGS19 or GNAI3 inhibited TNF-induced modification of LC3. Control and RGS19-knockdown (**C**) or GNAI3 knockdown (**D**) L929 cells were treated with mock or TNF (1 ng/ml) for 12 h. Cell lysates were subjected to western blot analysis with the indicated antibodies. (**E**) Knockdown of RGS19 or GNAI3 impaired zVAD-induced LC3 flux. Control and RGS19-knockdown or GNAI3-konckdown L929 cells were cultured with or without chloroquine (25 µM) and treated with or without zVAD (20 µM) for 12 h. LC3 levels were measured by Western blot.

Studies have shown that RGS19 and GNAI3 are engaged in controlling autophagy in human colon cancer HT-29 cells [17], [18]. Since zVAD-induced death of L929 cells was reported to be mediated by autophagy [8], we examined whether zVAD-induced autophagy was affected by RGS19 and GNAI3. As conjugation of microtubule-associated protein 1 light chain 3 (LC3) with phospholipids is considered as an autophagosome marker. We first analyzed modification of LC3-II in control and RGS19 knockdown cells that were treated with or without zVAD. zVAD-induced modification of LC3 (LC3-II) was indicated by the increase of faster migrated protein band on Western blot (Figure 2A). Knockdown of RGS19 inhibited zVAD-induced LC3 modification. As is reported previously [3], knockdown of RIP3, RIP1 or PI3KC3 all inhibited zVAD-induced increase of LC3-II. The effect of GNAI3 knockdown was similar to that of RGS19 knockdown (Figure 2B).

Since TNF has also been reported to induce autophagy, we then examined the role of RGS19 and GNAI3 in TNF-induced autophagy. Knockdown of RGS19 and GNAI3, as well as RIP1, RIP3, and PI3KC3 all inhibited TNF-induced modification of LC3 (Figure 2C, 2D).

Since the increase of LC3-II could also be resulted from blockage of autophagy at the degradation steps, we measured autophagic flux of zVAD-treated L929 cells to make sure that zVAD treatment leads to autophagy induction rather than inhibition of autophagy at the degradation steps. We used an autophagic flux assay based on the inhibition of LC3-II turnover. We treated the cells with or without lysosomotropic reagent chloroquine, which inhibits acidification inside lysosome and thus blocks LC3-II degradation. As anticipated, chloroquine led to an increase in LC3-II levels in all cell samples (Figure 2E). The LC3-II level in chloroquine plus zVAD treated L929 cells is higher than that in zVAD or chloroquine treated L929 cells (Figure 2E), indicating that zVAD induces LC3-II generation rather than blocks its degradation. The LC3-II level in RGS19 or GNAI3 knockdown cells treated with chloroquine+zVAD is the same as that treated with chloroquine alone, indicating that zVAD’s effect was eliminated by RGS19 or GNAI3 knockdown. These data demonstrate that RGS19 and GNAI3 are required for zVAD-induced autophagy.

### Deficiency in zVAD- and TNF-induced RGS19, GNAI3 and RIP3 Dependent Autophagy Eliminates zVAD- but not TNF-induced Cell Death in L929 Cells

**Figure 3:**
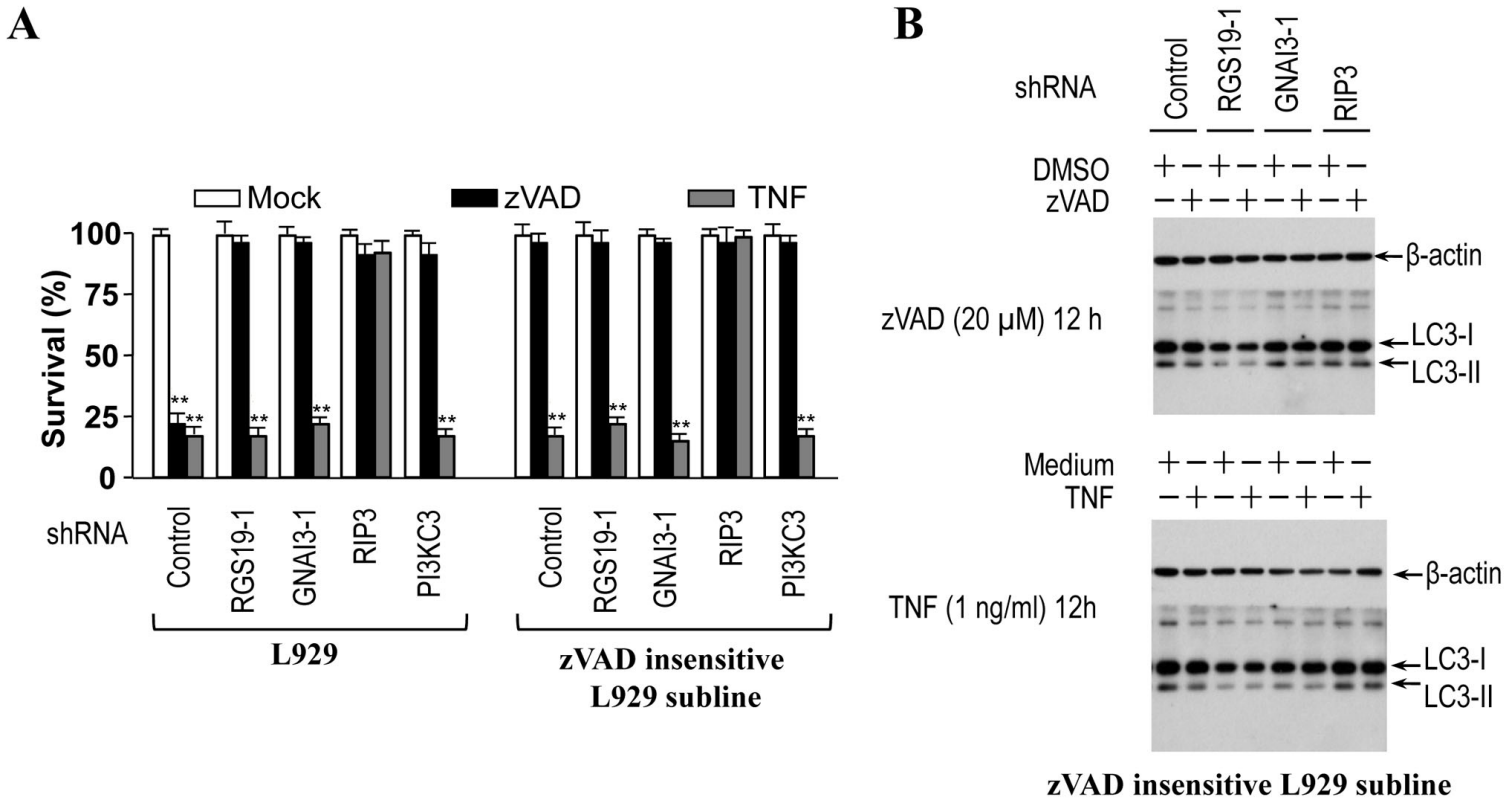
RGS19-, GNAI3- and RIP3-dependent autophagy is required for zVAD-induced but not TNF-induced cell death. (**A**) Knockdown of RGS19, GNAI3 or PI3KC3 had no effect on TNF-induced cell death in zVAD insensitive L929 subline. Control and RGS19- or GNAI3-knockdown L929 cells (left panel) and zVAD insensitive L929 cells (right panel) were treated for 24 h with mock, TNF (10 ng/ml) or zVAD (20 µM). Then cell viabilities were measured. (**B**) zVAD insensitive L929 subline had defect in RGS19-, GNAI3- and RIP3-dependent zVAD- or TNF-induced modification of LC3. Control and RGS19-, GNAI3- or RIP3-knockdown zVAD insensitive L929 cells were treated with mock, TNF (1 ng/ml) or zVAD (20 µM) for 12 h. Cell lysates were subjected to western blot analysis with antibodies against LC3 and β-actin. **, p<0.01.

We show in Figure 1B that knocking down PI3KC3, an essential mediator of autophagy, blocked zVAD- but not TNF-induced cell death. To further evaluate the role of autophagy in zVAD-induced cell death, we isolated a L929 subline that is not sensitive to zVAD-induced cell death (Figure 3A). The zVAD insensitive L929 subline responds to TNF normally in terms of cell death (Figure 3A). TNF-induced cell death of the zVAD insensitive cells is RIP3 dependent. Knockdown of RGS19, GNAI3 or PI3KC3 had no effect on TNF-induced cell death in this L929 subline. We analyzed LC3 modification in zVAD insensitive L929 cells and found that both zVAD and TNF cannot induce any change of the level of LC3-II (Figure 3B). Consistently, knockdown of RGS19, GNAI3 or RIP3 had no effect on LC3 modification in zVAD- or TNF-treated zVAD insensitive L929 cells, suggesting that the zVAD insensitive L929 subline has defect in RGS19, GNAI3 and RIP3 dependent autophagy response. This autophagy pathway in L929 cells is required for zVAD-, but not TNF-induced L929 cell death.

### Blocking Autophagy Pathway Inhibits zVAD-induced TNF Production

**Figure 4:**
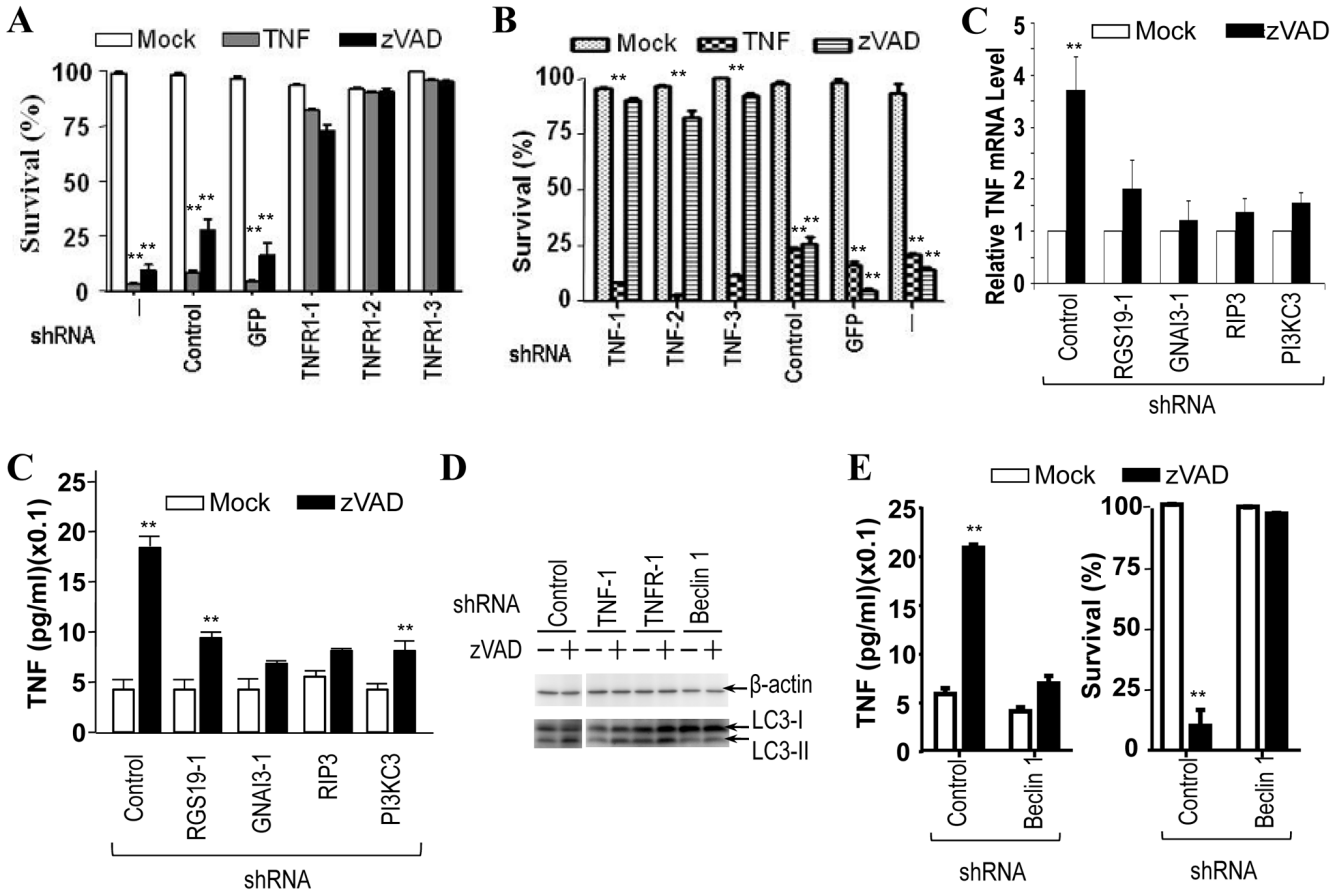
Blocking autophagy pathway inhibits zVAD-induced TNF production. (**A**) Knockdown of TNFR1 inhibited both TNF- and zVAD-induced cell deaths. Control and GFP- or TNFR1-knockdown L929 cells were treated with mock, TNF (10 ng/ml) or zVAD (20 µM) for 24 h. Then cell viabilities were measured. (**B**) Knockdown of TNF inhibited zVAD- but not TNF-induced cell death. Control and GFP- or TNF-knockdown L929 cells were treated with mock, TNF (10 ng/ml) or zVAD (20 µM) for 24 h. Then cell viabilities were measured. (**C**) Knockdown of RGS19, GNAI3, RIP3, or PI3KC3 all inhibited zVAD-induced increase of TNF mRNA. Control and RGS19-, GNAI3-, RIP3- or PI3KC3-knockdown L929 cells were treated with mock or zVAD (20 µM) for 3 h. TNF mRNA levels were measured by real-time PCR. (**D**) Knockdown of RGS19, GNAI3, RIP3, or PI3KC3 all inhibited zVAD-induced TNF secretion. Control and RGS19-, GNAI3-, RIP3- or PI3KC3-knockdown L929 cells were treated with mock or zVAD (20 µM) for 24 h. Then the cell culture medium was collected and concentrated 10 folds, and the TNF secretion was determined by ELISA. (**E**) Beclin-1, but not TNF or TNFR1, is required for zVAD-induced LC3 modification. Control and TNF-knockdown or TNFR-knockdown or Bclin 1-knockdown L929 cells were treated with mock or zVAD (20 µM) for 12 h. Cell lysates were subjected to Western blot analysis with antibodies against LC3 and β-actin. (**F**) Control and Bclin 1-knockdown L929 cells were treated with mock or zVAD (20 µM) for 12 h. Then TNF secretion was determined (left) and cell viabilities were measured (right). **, p<0.01.

It was reported that zVAD-induced L929 cell death depends on autocrine of TNF [13]. To investigate whether autocrine of TNF occurs in our experimental system, we used shRNAs to knock down TNF receptor 1 (TNFR1) or TNF and examined zVAD- and TNF-induced cell death in these knockdown cells. Knockdown of TNFR1 inhibited both TNF- and zVAD-induced cell deaths, whereas knockdown of TNF only inhibited zVAD-induced cell death (Figure 4A, 4B). Thus, zVAD-induced cell death does require autocrine production of TNF. We then examined zVAD-induced TNF production in L929 cells. Real-time PCR revealed an about three fold induction of TNF mRNA in L929 cells by zVAD treatment and the knockdown of RGS19, GNAI3, RIP3, or PI3KC3 all inhibit this induction (Figure 4C). Directly measuring the secreted TNF in the medium of zVAD-treated L929 cells cannot provide reliable information of zVAD-induced TNF production because the signals we detected by using the ELISA kit were around the basal line. However, we did detect zVAD-induced TNF secretion when the cell culture medium was concentrated to about 10 folds. Knockdown of RGS19, GNAI3, RIP3, and PI3KC3 all inhibited zVAD-induced TNF production (Figure 4D), suggesting that zVAD-induced autophagy response is involved in zVAD-induced TNF production.

To further analyze whether the TNF secreted by zVAD-treated L929 cells plays any role in zVAD-induced autophagy, we analyzed zVAD-induced increase of LC3-II in TNF knockdown and TNFR1 knockdown cells. Beclin-1 knockdown L929 cells were included as a control of autophagy defective cells. TNF knockdown and TNFR1 knockdown did not have much effect on zVAD-induced increase of LC3 modification (Figure 4E). As anticipated, Beclin-1 knockdown blocked zVAD-induced increase of LC3-II (Figure 4E). Beclin-1 knockdown also inhibited zVAD-induced TNF secretion and cell death inL929 cells (Figure 4F). Collectively, our data indicate that zVAD-induced autophagy is involved in zVAD-induced TNF production.

### Inhibition of ERK- or JNK- Pathway Blocks the zVAD-, but not TNF-, Induced Cell Death

**Figure 5:**
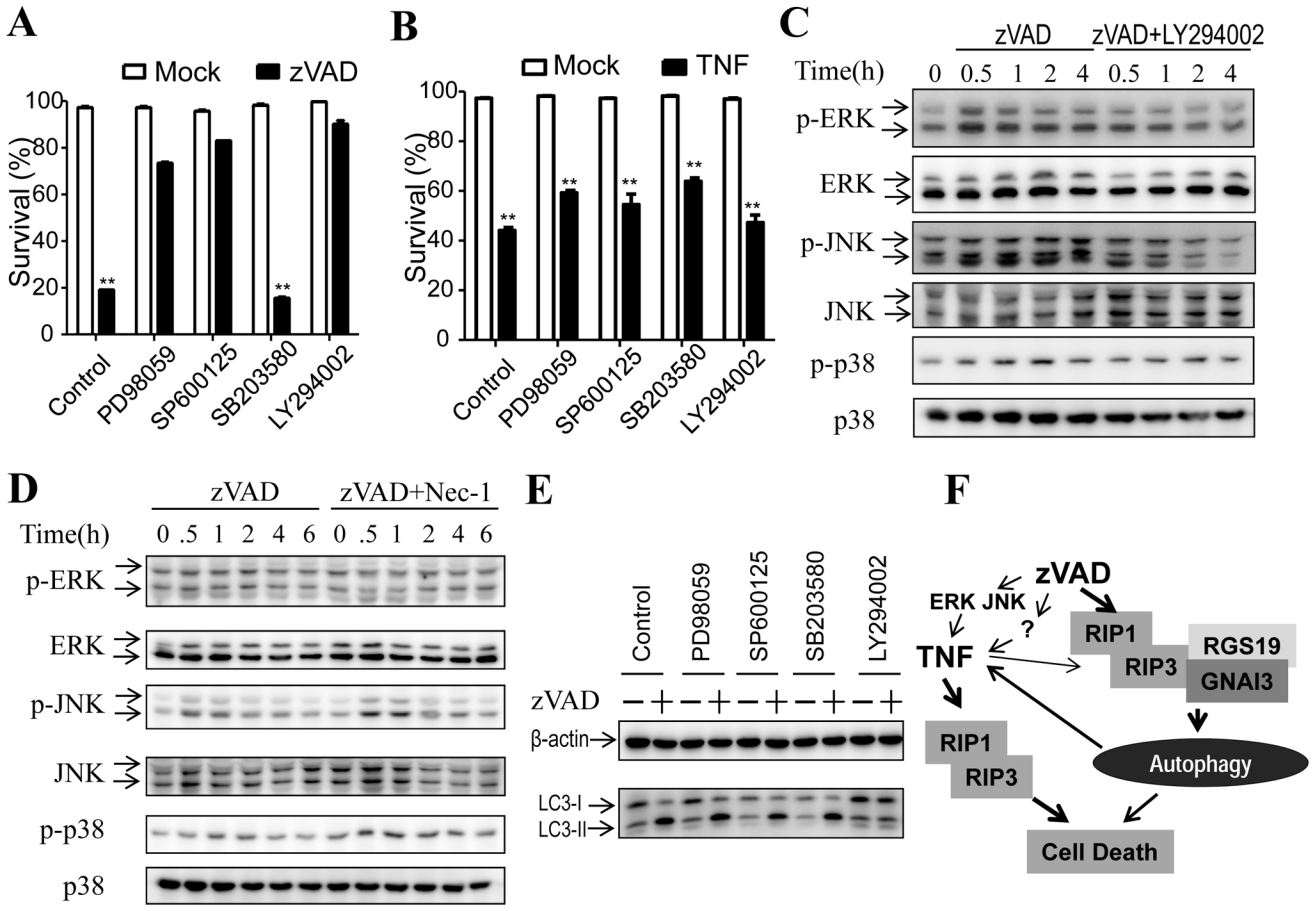
Inhibition of ERK- or JNK- pathway blocks zVAD- but not TNF-induced cell death. (**A**) Inhibition of ERK- or JNK- pathway blocked zVAD-induced cell death. L929 cells were untreated or treated with ERK inhibitor PD98059 (100 µM), JNK inhibitor SP600125 (10 µM), p38 inhibitor SB203580 (10 µM), and PI3K inhibitor LY294002 (100 µM), respectively. Then the cells were treated with mock or zVAD (20 µM) for 24 h and cell viabilities were measured. (**B**) Inhibition of MAPKs does not block TNF-induced cell death. L929 cells were untreated or treated with PD98059 (100 µM), SP600125 (10 µM), p38 inhibitor SB203580 (10 µM), and LY294002 (100 µM). Then the cells were treated with mock or TNF (10 ng/ml) for 24 h and cell viabilities were measured. (**C**) zVAD had little effect on MAPKs’ activation. L929 cells were treated with zVAD or zVAD+LY294002 for 0, 0.5, 1, 2, 4 h, respectively. Cell lysates were subjected to western blot analysis with antibodies against p-ERK, ERK, p-JNK, JNK, p-p38 and p38. (**D**) Inhibition of zVAD-induced cell death has no effect on MAPKs’ activation. L929 cells were treated with zVAD or zVAD+Nec-1 for 0, 0.5, 1, 2, 4, 6 h, respectively. Cell lysates were subjected to western blot analysis with the indicated antibodies. (**E**) Inhibitors of MAPKs did not affect zVAD-induced LC3 modification. L929 cells were untreated or treated with PD98059 (100 µM), SP600125 (10 µM), SB203580 (10 µM), or LY294002 (100 µM). Then the cells were treated with mock or zVAD (20 µM) for 12 h and LC3 were analyzed by western blot. (**F**) Hypothetical pathway network of zVAD-induced cell death. The main pathways of zVAD-induced cell death are autophagy and autocrine of TNF. The induction of TNF by zVAD may depend on the collective effect of a number of pathways such as ERK, JNK, and autophagy. Mechanisms of zVAD-induced L929 cells could be different due to the variation of L929 sublines. **, p<0.01.

A study showed that MAPKs pathway is required for zVAD-induced TNF production and TNF in turn causes cell death [13]. Another report indicated that zVAD induces c-Src dependent activation of MAPKs which leads to the generation of reactive oxygen species that causes cell death [10]. Although these two studies suggested different mechanisms of zVAD-induced L929 cell death, both of them showed the involvement of MAPKs in zVAD-induced cell death. Therefore, we tested whether inhibition of ERK, JNK, or p38 by chemical inhibitors would affect zVAD-induced cell death in our experimental condition. The addition of MEK inhibitor PD98059 (which inhibits ERK- pathway) or JNK inhibitor SP600125 inhibited zVAD-induced cell death, while p38 inhibitor SB203580 did not inhibit zVAD-induced cell death (Figure 5A). However, none of these inhibitors had effect on TNF-induced cell death (Figure 5B). We also included PI3K inhibitor LY294002 in the experiment and observed its inhibitory effect on zVAD- but not TNF-induced cell death.

Because zVAD-induced autophagy pathway is required for TNF production (Figure 4C, 4D, 4F) and activation of ERK and JNK by zVAD is required for TNF production [13], we ought to check the relationship between MAPKs and autophagy pathway. We examined zVAD’s effect on MAPKs’ activation by measuring the phosphorylation of ERK, JNK and p38. zVAD only had slight effect on ERK, JNK, and p38 phosphorylation in our experimental system and PI3K inhibitor LY294002 also had no or very little effect on the phosphorylation of ERK, JNK, and p38 (Figure 5C). We also tested Necostatin-1 (Nec-1), an inhibitor of RIP1 that can block RIP1-RIP3 signaling and inhibit zVAD-induced cell death [13], and found it had no effect on ERK, JNK, or p38 phosphorylation (Fig. 5D). We also analyzed the effect of the inhibition of ERK, JNK, or p38 on zVAD-induced increase of LC3-II and found that these MAP kinases play no role in zVAD-induced autophagy (Figure 5E). All together, our data showed that zVAD-induced activation of ERK, JNK, or p38 is very modest; zVAD-activated autophagy pathway has no effect on MAPK pathways and the MAPKs play no role in zVAD-induced autophagy.

## Discussion

zVAD-induced autophagy-related cell death in L929 cells was observed nearly ten years ago [8]. ATG7 and beclin 1, the key proteins of autophagy pathway, were found to be activated by caspase inhibition, which linked autophagy to RIP1 and JNK activation that was believed to trigger the cell death. Since then, zVAD-induced L929 cell death was considered as a model of autophagy-mediated cell death. However, Wu et al reported later that autophagy did not promote but rather played a protective role in zVAD-induced L929 cell death in their experimental system [11]. It is clear that experimental conditions could influence the outcome of the experiments since we confirmed Yu’s result that zVAD-induced autophagy had promoting but not inhibiting effect on zVAD-induced L929 cell death in our experimental system. We showed here that RGS19 and its partner GNAI3 function with RIP1 and RIP3 to promote autophagy and are involved in zVAD-induced cell death. We also confirmed Wu’s data that zVAD indeed induces a small amount of TNF production and the autocrine effect of TNF is required for zVAD-induced cell death (Fig. 4). We were able to reproduce Wu’s data that starvation of cells or treatment of the cells with rapamycin can suppress zVAD-induced L929 cell death (Figure S1). However, we found that neither starvation nor rapamycin treatment can induce autophagy in L929 cells (Figure S2), suggesting that the effect of starvation and rapamycin on zVAD-induced cell death in our experimental system is not related to autophagy induction. The cell type dependence of starvation- or rapamycin-induced autophagy has been reported [22], The L929 cells in our laboratory should belong to the cell types that are insensitive to these two classic autophagy inducers. There are several mTOR-independent autophagy induction pathways [23], [24], [25], JNK1/Beclin-1/PI3KC3, an mTOR-independent pathway could be involved in zVAD-induced autophagy in L929 cells. We also observed enhancing effect of chloroquine on zVAD-induced cell death as Wu’s group did (Figure S1), suggesting that inhibition of degradation steps of autophagosome could enhance zVAD-induced cell death but the mechanism of which is unclear.

It is very interesting to note that in L929 cells zVAD-induced cell death requires the signaling of both autophagy and TNF while TNF-induced cell death does not need autophagy. Since we have performed these experiments side by side, this result cannot be due to variation of experimental conditions but intrinsic mechanisms. When the cells were cultured in six-well plate with ∼70% confluence, zVAD-induced TNF in the medium was about a few pgs per ml, whereas the concentration of TNF used in triggering cell death has to reach ng level per ml in the absence of zVAD (data not shown). This explains that cell death induced by TNF at a concentration >1 ng/ml does not need the help of autophagy pathway. However, zVAD-induced TNF production by itself is not sufficient to induce cell death, thus the promoting effect of autophagy and inhibition of caspase by zVAD appear to be required too.

The activation of ERK and JNK by zVAD in L929 cells were reported independently by two groups, although the activation mechanisms were proposed differently in their publications [10], [13]. We showed that inhibition of ERK or JNK blocked zVAD-induced L929 cell death, however, we only observed little or no activation of these MAPKs, suggesting that basal activities of MAPKs are required for zVAD-induced cell death in our experimental conditions. We think the significant activation of ERK and JNK by zVAD observed by Wu et al [13] is most likely due to that their L929 cell line is somehow different from ours, since we all know that cell line can change after long time culture under different conditions. Nonetheless, some of the data we have are the same as Wu’s. For example, we did observe the same result that zVAD can induce TNF production in L929 cells and the induced TNF is required for L929 cell death.

It appears that the mechanisms of zVAD-induced L929 cell death could be different due to the variation of different L929 sublines. However, the main pathways that involved in zVAD-induced cell death should be autophagy and autocrine of TNF. In certain L929 cells cell death requires cooperation of autophagy and TNF production induced by zVAD. In some other L929 sublines the autocrine effect is sufficient to induce cell death. The induction of TNF by zVAD may depend on the collective effect of a number of pathways including ERK, JNK and autophagy, and the contributions of these pathways in TNF production could be distinct in different L929 sublines. And the zVAD-induced autophagy depends on RGS19/GNAI3 as well as RIP1/RIP3. Based on current data, we propose a pathway network of zVAD-induced cell death shown in Figure 5F, and suggest that different L929 sublines might preferentially rely on one pathway more than others.

## Supporting Information

Figure S1 **The effect of starvation of the cells, rapamycin or chloroquine-treatment on zVAD-induced cell death in L929 cells.** Cell viabilities were measured of the L929 cells that were cultured under starvation for 12 h and then treated with zVAD for 24 h; pre-treated with rapamycin for 30 min and then treated with zVAD for 24 h; pre-treated with chloroquine for 30 min and then treated with zVAD for 24 h; and mock treated or treated with zVAD for 24 h.
(TIF)

Figure S2 **The L929 cell line used in this study is not sensitive to classic autophagy induction.** Control and RGS19-knockdown or GNAI3 knockdown L929 cells were cultured with or without chloroquine under starvation or rapamycin treatment for 12h. LC3 levels were measured by western blot.
(TIF)
