# Supplementary figures and images for: Regulator of G-Protein Signaling 19 (RGS19) and Its Partner Gα-Inhibiting Activity Polypeptide 3 (GNAI3) Are Required for zVAD-Induced Autophagy and Cell Death in L929 Cells
Source: PLoS One. 2014 Apr 21;9(4):e94634. doi: 10.1371/journal.pone.0094634 (PMC3994006; doi:10.1371/journal.pone.0094634)

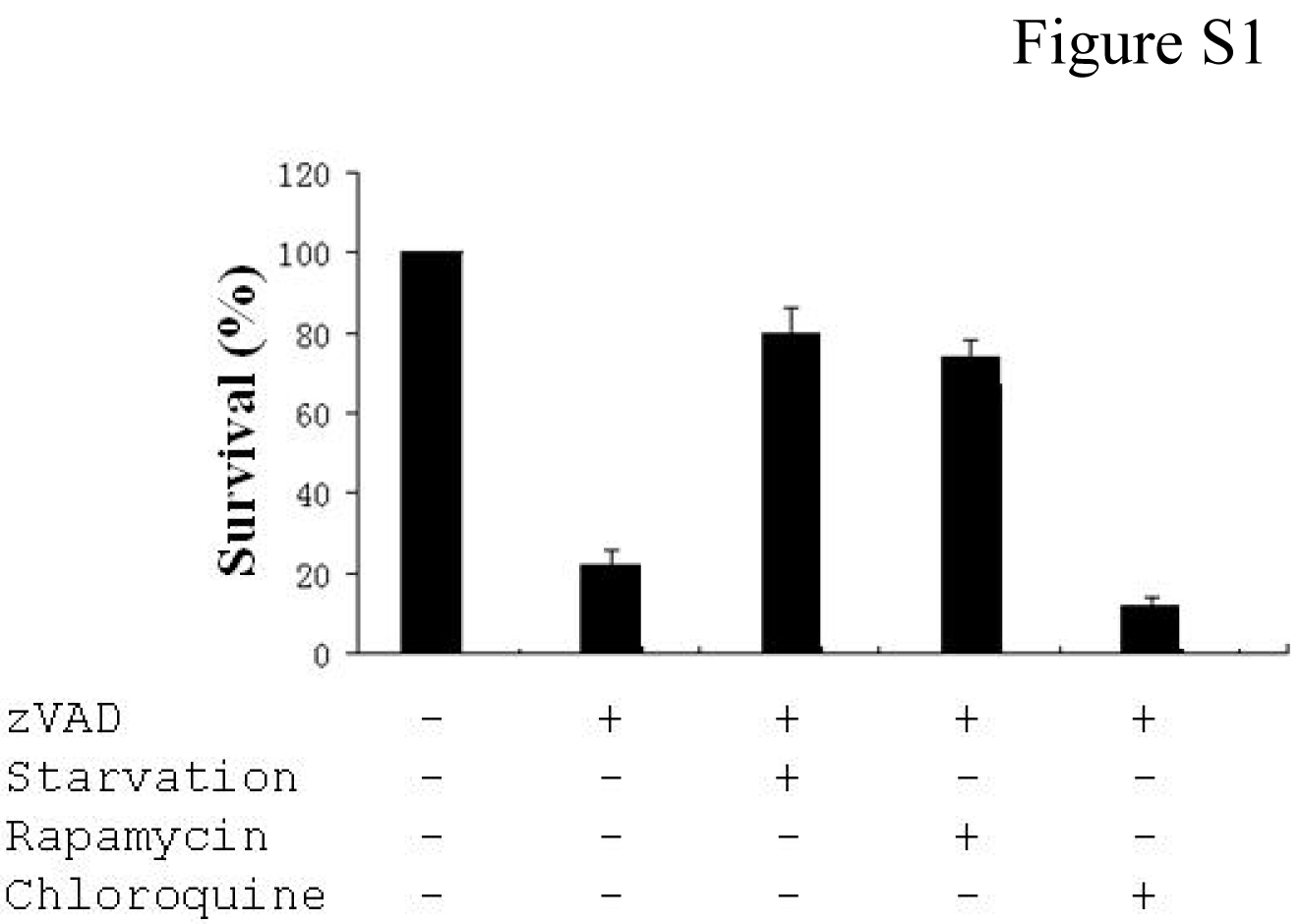

Supplement: Figure S1 — The effect of starvation of the cells, rapamycin or chloroquine-treatment on zVAD-induced cell death in L929 cells. Cell viabilities were measured of the L929 cells that were cultured under starvation for 12 h and then treated with zVAD for 24 h; pre-treated with rapamycin for 30 min and then treated with zVAD for 24 h; pre-treated with chloroquine for 30 min and then treated with zVAD for 24 h; and mock treated or treated with zVAD for 24 h. (TIF) [file pone.0094634.s001.tif]

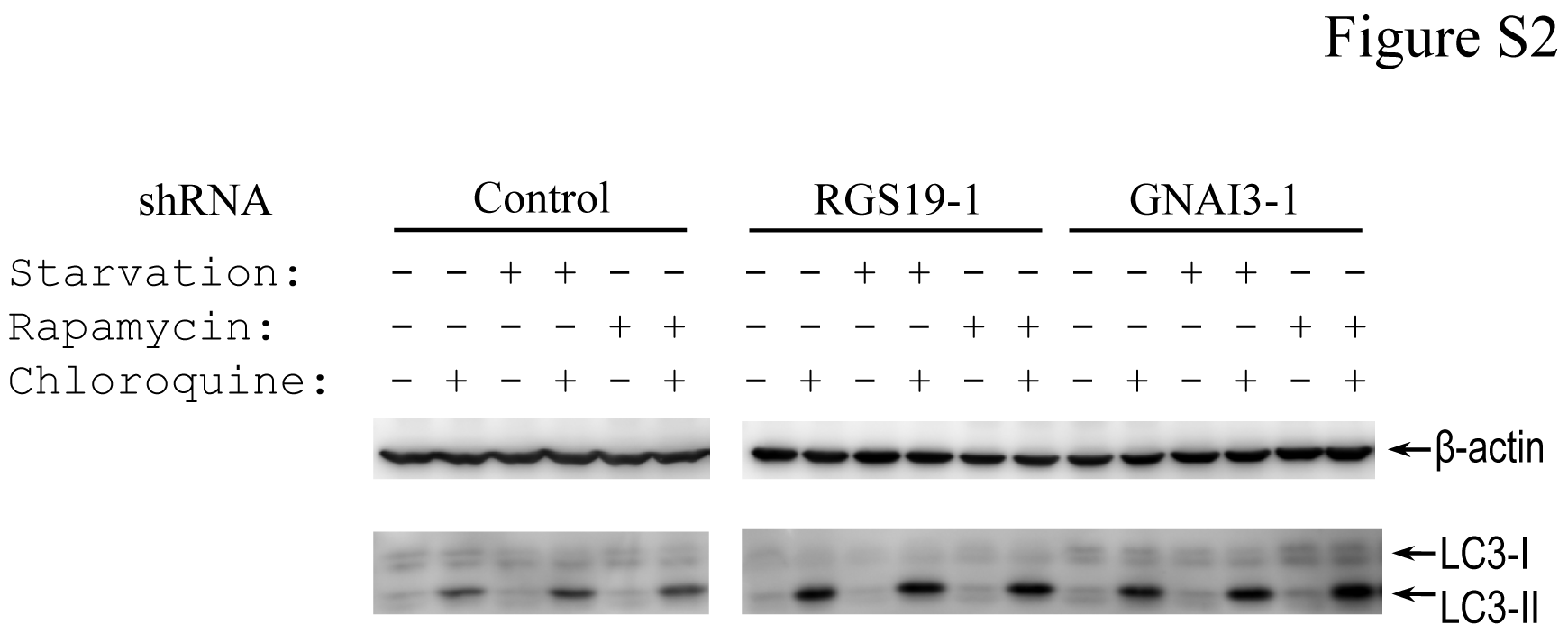

Supplement: Figure S2 — The L929 cell line used in this study is not sensitive to classic autophagy induction. Control and RGS19-knockdown or GNAI3 knockdown L929 cells were cultured with or without chloroquine under starvation or rapamycin treatment for 12h. LC3 levels were measured by western blot. (TIF) [file pone.0094634.s002.tif]
